# Evaluation of machine learning models that predict lncRNA subcellular localization

**DOI:** 10.1093/nargab/lqae125

**Published:** 2024-09-18

**Authors:** Jason R Miller, Weijun Yi, Donald A Adjeroh

**Affiliations:** Department of Computer Science and Information Technology; Hood College, Frederick, MD 21701, USA; Lane Department of Computer Science and Electrical Engineering; West Virginia University, Morgantown, WV 26506, USA; Lane Department of Computer Science and Electrical Engineering; West Virginia University, Morgantown, WV 26506, USA; Lane Department of Computer Science and Electrical Engineering; West Virginia University, Morgantown, WV 26506, USA

## Abstract

The lncATLAS database quantifies the relative cytoplasmic versus nuclear abundance of long non-coding RNAs (lncRNAs) observed in 15 human cell lines. The literature describes several machine learning models trained and evaluated on these and similar datasets. These reports showed moderate performance, *e.g*. 72–74% accuracy, on test subsets of the data withheld from training. In all these reports, the datasets were filtered to include genes with extreme values while excluding genes with values in the middle range and the filters were applied prior to partitioning the data into training and testing subsets. Using several models and lncATLAS data, we show that this ‘middle exclusion’ protocol boosts performance metrics without boosting model performance on unfiltered test data. We show that various models achieve only about 60% accuracy when evaluated on unfiltered lncRNA data. We suggest that the problem of predicting lncRNA subcellular localization from nucleotide sequences is more challenging than currently perceived. We provide a basic model and evaluation procedure as a benchmark for future studies of this problem.

## Graphical Abstract

**Graphical Abstract:**
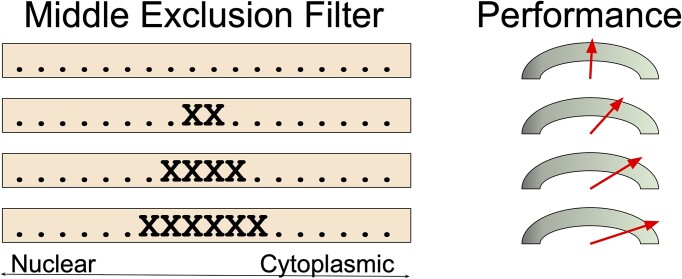

## Introduction

The human genome contains about 20K protein-coding genes and possibly as many long non-coding RNA (lncRNA) genes. The latter are defined as genes whose RNA transcripts of over 200 nucleotides are not translated to proteins. The majority of lncRNA genes have unknown functions, but the RNA transcripts of several lncRNA genes have been shown to play important roles in cellular biology and human health (1,2).

To gain insight into the functions of uncharacterized lncRNA genes, researchers have measured the abundances of lncRNA molecules in different compartments of cells (1). The ENCODE project sequenced RNA from nuclear and cytosolic components of 15 human cell lines (3–6). The RNA-seq reads were assigned to genes by aligning them to the reference human genome. After normalizations, the nuclear and cytosolic relative abundance was determined per cell line per gene. These results were presented as the lncATLAS database (7), which offers a derived metric called the cytoplasmic-to-nuclear relative concentration index (CNRCI). The database gives a CNRCI value for several hundred to several thousand lncRNA genes per cell line. For selected genes with known functions (e.g. MALAT1 and DANCR), the CNRCI values were consistent with biological knowledge (7). These consistencies suggested that the CNRCI values could assist functional characterization of as-yet uncharacterized lncRNA genes.

Several machine learning studies used the ENCODE or lncATLAS data to predict whether abundance is higher in the cytoplasm or nucleus. They each trained models on some large portion of the available data and evaluated on the remainder. Though the database labels were quantitative, these models were trained to generate binary labels representing cytoplasmic or nuclear preference. The inputs to these models included the nucleotide sequences of reference transcripts for each gene. The sequences were taken from the GENCODE database which provides canonical DNA and RNA sequences for the human reference (8–11). The use of nucleotide sequences as predictor variables was sensible, since subcellular localization had been correlated with certain sequence features (called k-mers *i.e*. substrings) for some lncRNAs (12). Some of the models incorporated other predictor features, such as the lncRNA gene’s chromosome and whether the gene was antisense to a protein-coding gene (13).

At least four groups have published models using the quantitative abundance data from ENCODE or lncATLAS and the RNA sequence data from GENCODE. Gudenas & Wang (13) described a three-layer multilayer perceptron called **DeepLncRNA** whose inputs consisted of k-mer frequency profiles of each gene’s canonical RNA transcript sequence, plus gene features provided by GENCODE. The authors reported 72.4% accuracy and 78.7% AUROC (area under receiver operating characteristic) on their test set derived from ENCODE. Lin et al. (14) described a neural network called **lncLocator 2.0** which had embedding, convolutional, recurrent and fully-connected layers. Its inputs consisted of each gene’s RNA transcript sequences. The authors reported up to 84.7% AUROC on an individual cell line, using their test set derived from lncATLAS. Jeon et al. (15) described an ensemble model called **TACOS** that ‘consistently outperformed lncLocator 2.0’ on their test set. Yuan et al. (16) described a light gradient boosting machine called **RNAlight**. Its inputs consisted solely of k-mer frequency profiles, k∈{3,4,5} of each gene’s canonical RNA transcript sequence. The authors reported 72% accuracy on their main lncRNA test set. Recently, Liu et al. (17) presented a lncLocator extension called **lncLocator-imb** and having ‘superior performance’ to ‘previous state-of-the-art’ on several types of RNA including lncRNA.

Each study incorporated a data preparation protocol that we call ‘middle exclusion.’ Lin *et al.* described it this way: ‘In order to make it easier for classification, we filter out the data with CNRCI in [-1,1]. Thus, the preserved data, with CNRCI larger than 1 or smaller than -1, are significantly located in cytoplasm or nucleus.’ Though some studies used different thresholds, all the studies excluded about half their data while retaining the upper and lower quartiles. In each study, the middle exclusion filter was applied prior to designating the training and testing portions. As a result, the training and testing subsets both consisted of extreme cases only. Thus, the performance statistics reported by these studies reflect a very special situation in which the model is used to predict the localization of genes whose compartmental abundances have already been characterized to exclude the middle values. We explored the effect of the middle exclusion protocol on machine learning performance statistics.

In each localization study, a machine learning model was designed and parameterized through frequent consultation with a training set, then finally evaluated on separate data that had been withheld from training. Consultation of the test set during training is known to allow information leakage, a procedural ‘flaw in machine learning that leads to overoptimistic results’ (18). A leak can ‘occur when the training set is processed in a manner that depends on data from the test set’ (19). Reviews have described several pitfalls that could induce information leakage in genomics (19) and actual examples from 294 published studies across 17 fields of science (18). We examined the localization studies for indications of whether their performance statistics could have been influenced by information leakage.

Finally, we describe an evaluation protocol and demonstrate it on a simple model. This binary classifier labels lncRNA as nuclear or cytoplasmic based on the k-mer profiles of the RNA sequences. Though the model’s performance statistics fall short of those previously published, we offer the model and the protocol as an appropriate benchmark against which other classifiers may be compared.

## Materials and methods

Subcellular abundance data for 15 human cell lines was downloaded in CSV format from the lncATLAS database (7); see Supplement 1. RNA sequences for the human reference were downloaded in FASTA format from GENCODE (11); specific experiments used either version 44 or 45 as apparent from the filenames. The canonical transcript per gene was selected based on the ‘Ensembl_canonical’ tag in the GENCODE annotation in GFF3 format; see Supplement 2. The RNAlight source code and data were obtained from the URL cited in that paper (16) and the LightGBM code was obtained from the URL cited in that paper (20); see Supplement 3. The lncLocator 2.0 source code and benchmark data were obtained from the URLs cited in that paper (14); see Supplement 4. The TACOS web server was accessed at the URL cited in that paper (15); see Supplement 5. The DeepLncRNA data was obtained from the supplement of that paper (13); see Supplement 6.

Data processing and machine learning programs were written in Python 3.10 or higher and run inside Jupyter notebooks. The classical machine learning models [random forest (RF), gradient boosting, support vector] were implemented with the scikit-learn library (21). Long-running programs used virtual computers at Google CoLab, sometimes with an A100, V100 or L4 GPU; see Supplement 7. The benchmark was created from lncATLAS and GENCODE data using a LightGBM model (20) with code adapted from the RNAlight repository; see Supplement 8. All the notebooks are available online and described in the supplement.

## Results

### Four studies selected for analysis

Our literature survey identified machine learning studies that relied on quantitative lncRNA subcellular abundance data such as that provided by the ENCODE or lncATLAS resources. The survey identified these four localization studies for review: Gudenas & Wang (13), Lin *et al.* (14), Jeon *et al.* (15) and Yuan *et al.* (16). The first used ENCODE directly, while the others obtained ENCODE data from lncATLAS. All these studies used RNA sequences from the GENCODE database. These studies are summarized in Table 1. A fifth study (17) presented an extension of Lin *et al.* while using similar data and protocols.

**Table 1.** Summary of the data preparation protocols used in the four studies that applied machine learning to quantitative lncRNA localization data derived from human cell lines

	Model, authors	Data preparation summary
**A**	RNAlight Yuan *et al.* (16)	This study used lncATLAS CNRCI values, which are base-2 logs of cytoplasmic-to-nuclear (C-to-N) normalized abundance ratios. Each lncRNA gene was characterized by its mean CNRCI value across 14 of the 15 cell lines. Genes having CNRCI < -2 were labeled cytoplasmic and genes having CNRCI > 0 were labeled nuclear. Genes with intermediate values, constituting about 39%, were excluded. The classes were approximately balanced with 1525 cytoplasmic and 1983 nuclear genes. Data from two smaller databases were added using thresholds chosen to exclude their middle values. Genes were then filtered if their indicated subcellular compartments differed between cell lines or between data sources.
**B**	lncLocator2.0 Lin *et al.* (14)	This study used lncATLAS CNRCI values for 15 cell lines. Each cell line was analysed separately. Class imbalance varied per cell line. Genes having CNRCI < -1 were labeled nuclear and genes having CNRCI > +1 were labeled cytoplasmic. These thresholds exclude intermediate values, affecting 29%–52% of genes per cell line.
**C**	TACOS Jeon *et al.* (15)	This study used lncLocator 2.0 data for 10 of the 15 cell lines. To balance the training subsets, samples were shifted from the training subsets to the validation or testing subsets for each cell line.
**D**	DeepLncRNA Gudenas & Wang (13)	This study used the ENCODE data directly (without lncATLAS). A nuclear-to-cytoplasmic (inverse of the CNRCI) log2 fold-change (L2FC) was computed using data for 14 human cell lines. From all the characterized genes, the upper and lower quartiles were selected. Genes having L2FC < 0 were labeled cytoplasmic and genes having L2FC > 2.8 were labeled nuclear. Genes with middle values were excluded from the study. This scheme generated two opposite and balanced classes with about 4300 genes per class. About 8600 genes (half) were excluded.

Our survey excluded the SEEKR study (12) because it did not present a predictive model. Its analysis of the ENCODE and GENCODE data clustered genes based on transcript k-mer content using k = 6 for the main study. The study found that clusters with different k-mer profiles had different localization profiles in two human cell lines. Of note, the study used a precursor to the middle exclusion filter by identifying specific k-mers that were enriched within the most nuclear or the most cytoplasmic lncRNAs.

Our survey excluded studies that relied on the qualitative labels provided by the RNAlocate (22,23) database, for which middle exclusion is not possible. RNAlocate encompasses a variety of species, RNA classes and cellular compartments, with data compiled from literature searches and data curation. Quantitative abundance data is available online for some genes, but these details are omitted from query results and bulk downloads. Common data filters require a certain evidence type or a minimum sample size. Tools trained on RNAlocate data include the first lncLocator (24) as well as iLoc-lncRNA (25), GM-lncLoc (26), DeepLncLoc (27), LightGBM-LncLoc (28), GraphLncLoc (29), ncRNALocate-EL (30), LncLocFormer (31) and EL-RMLocNet (32). Their performance is addressed in Discussion.

Our survey excluded a predictive model that relied on an entirely different set of RNA features, namely splicing efficiency measurements taken on ENCODE cell lines (33). This study reported precision and recall values above 60% for lncRNAs.

### Middle exclusion effect

We questioned what effect the middle exclusion filter has on machine learning. In the selected studies, the filter was applied to the full dataset before the training and testing partitions were created. Thus, the performance statistics in each study were derived from tests on filtered data. We wondered whether performance metrics based on extreme data would be predictive of model performance on intermediate data. Our first attempt to address this question used predictions made by the model builders. The results are summarized in Table 2.

**Table 2.** Evaluation of model performance on genes with intermediate abundance ratios. The gene represented here had been excluded from the training and evaluation subsets of their respective studies. (**A**) The RNAlight repository includes a file of predictions for all canonical lncRNA transcripts. The accuracy of binary labels was tabulated across those transcripts having a mean CNRCI value in lncATLAS. (**B**) Code and data were obtained from the lncLocator 2.0 repository. The code was modified to apply middle exclusion to the training subset but the inverse filter to the testing subset for the default 100 epochs. The experiment was run on the H1.hESC cell line which that study used as a baseline. (**C**) The TACOS web server was given 10 sequence files, one for each of its 10 supported cell lines. Each file represented canonical transcripts of randomly selected genes having an intermediate CNRCI value for that cell line. (**D**) It was not feasible to perform a similar test on DeepLncRNA. The data developed for the study, which did not use lncATLAS, were provided in the online supplement without the genes filtered by middle exclusion

	Model, authors	Model performance on excluded genes
**A**	RNAlight Yuan *et al.* (16)	55% accuracy was computed from a file of predictions on all genes, available in the source code repository.
**B**	lncLocator 2.0 Lin *et al.* (14)	59% accuracy was obtained on the ‘baseline’ cell line by training the model with middle exclusion and evaluating on middle genes from the test set.
**C**	TACOS Jeon *et al.* (15)	56% accuracy was obtained after submitting 100 randomly selected sequences for each of the 10 supported cell lines, 1000 total.
**D**	DeepLncRNA Gudenas & Wang (13)	N/A. The filtered portion of the data was not included in the supplemental data files.

RNAlight was reported to have 72% accuracy on lncRNA (16), using average CNRCI across 14 cell lines. To assess its performance on the excluded genes, we analysed a file in its source code repository that has RNAlight predictions for 15K human lncRNA transcripts. Most of these transcripts are not characterized in lncATLAS and a few were not found in the current GENCODE, but we matched 5313 transcripts to genes in GENCODE that have CNRCI values in lncATLAS. For those, we computed the mean CNRCI values and found 2091 whose values qualified for exclusion following the RNAlight protocol. Comparing the predictions for these middle genes to their lncATLAS values, we observed 55% accuracy; see Supplement 3. This is substantially lower than the 72% accuracy reported. Thus, RNAlight’s measured accuracy at predicting localizations for uncharacterized genes could range from 72% (for genes outside the middle) to 55% (for genes in the middle), but the portion of genes in the middle would be unknown. This exploration shows that *the reported accuracy of RNAlight is likely not a reliable predictor of its accuracy on uncharacterized lncRNA datasets*.

The lncLocator 2.0 model was trained and evaluated on each cell line separately. The cell line with the most CNRCI values, H1.hESC, was used for its baseline comparisons and on this cell line, AUROC was reported as 0.8472 (14). We ran lncLocator 2.0 code on the lncLocator benchmark data. We modified the code slightly to be able to test it with and without middle exclusion using H1.hESC. In a run that trained and tested the model with middle exclusion, we saw AUROC 0.8282 and 75% accuracy on the test set. But using the test set complement (*i.e*. only the genes that were excluded previously), we saw AUROC 0.5921 and 59% accuracy; see Supplement 4. Thus, if this model were used to make predictions on uncharacterized genes, for which middle exclusion could not be applied, the model’s performance would likely fall below the published level. This exploration shows that *the reported performance of lncLocator 2.0 is likely not a reliable predictor of its performance on uncharacterized lncRNA datasets*.

‘TACOS consistently outperformed lncLocator 2.0,’ according to its publication(15). We submitted selected sequences to the TACOS web server. For each of the 10 supported cell lines, we submitted canonical transcript sequences for 100 genes chosen randomly but having CNRCI values in the [-1,1] range for that cell line. The server was 56% accurate on these data; see Supplement 5. Thus, as with the previous models, *the performance of TACOS is likely to be less than claimed since uncharacterized datasets are likely to contain mid-range genes*.

The published DeepLncRNA performance statistics included 72% accuracy and 0.787 AUROC (13). Unfortunately, it was not feasible for us to measure this model on middle genes. This study developed its own dataset, with different features, by a complex process and provided only the filtered portion in its data supplement; see Supplement 6. Nevertheless, its performance statistics were clearly derived from test sets that excluded genes with middle values.

### Middle exclusion and model architectures

We explored whether the middle exclusion effect is specific to certain machine learning model architectures or is a general phenomenon across different architectures.

RNAlight (16) is a LightGBM (20) model with hyperparameters optimized on the RNAlight training subset; see Supplementary Table S2. We trained and evaluated a standard LightGBM implementation with its default parameters using RNAlight code for feature extraction and training. Table 3 shows the model’s performance on two datasets; see Supplement 3. The RNAlight dataset used in column A consists of data from lncATLAS data plus smaller contributions from other databases, minus genes with contradictory values across these sources. (This dataset is available in its filtered form, precluding any test without middle exclusion.) We built the dataset used in column B from lncATLAS data following the RNAlight protocol of averaging CNRCI values across 14 cell lines. Column C summarizes a test of the model without middle exclusion, that is, using all the available data. The metrics in columns A and B are similar, but the metrics in column C are substantially degraded, indicating that middle exclusion had boosted the metrics in the previous columns. For example, the LightGBM accuracy was 71% with middle exclusion and 61% without, on the same dataset. The above experiment was repeated on mRNA data and the results show similar middle exclusion effects; see Supplementary Table S6. Clearly, *our test using middle exclusion provided an optimistic view of LightGBM performance* as compared to its performance on unfiltered data.

**Table 3.** Middle-exclusion effect on a LightGBM model classifying lncRNA localization. (**A**) Performance of a LightGBM model using default hyperparameters tested on the RNAlight lncRNA dataset formed from lncATLAS with other data. (**B**) The LightGBM model performed similarly when trained and tested on lncATLAS data prepared by RNAlight protocols, including middle exclusion. (**C**) The LightGBM model was trained and tested on the lncATLAS data with its middle restored. The lesser performance metrics indicate that middle exclusion had boosted metrics in the previous columns. The cytoplasmic-versus-nuclear CNRCI threshold was -1. The statistics reflect two rounds of 5-fold cross-validation on 90% of the data (10% withheld for testing but not used here)

	A	B	C
Data treatment:	Middle exclusion	Middle exclusion	None
Data source:	RNAlight	lncATLAS	lncATLAS
Model:	LightGBM	LightGBM	LightGBM
**Accuracy**	67 ± 1	71 ± 3	61 ± 2
**F1**	69 ± 1	70 ± 2	60 ± 2
**AUPRC**	74 ± 1	78 ± 1	66 ± 3
**MCC**	0.347	0.414	0.227
**AUROC**	73 ± 1	78 ± 2	66 ± 2

Random forest (RF) (34), gradient boosting machines (GBM) (35) and support vector machines (SVM) (36) are classical machine learning algorithms with fundamentally different architectures. We tested middle exclusion on these models using standard implementations and default hyperparameters (21). We measured performance by cross-validation on the lncATLAS data which was prepared as above. We trained and tested each model with and without middle exclusion; see Supplement 7. As shown in Table 4, performance statistics were universally higher using the middle exclusion data than on the full dataset. For example, the AUROC of the RF model was 75% with middle exclusion but 66% without. These results show that *middle exclusion can boost performance statistics across a variety of machine learning architectures*.

**Table 4.** The middle-exclusion filter effect on performance metrics. Three classical models were each trained and tested with and without the middle exclusion filter applied to the full dataset. The results reflect two rounds of 5-fold cross-validation on 90% of the data (10% withheld for testing but not used here)

Model:	RF		GBM		SVM	
Filter:	Middle exclusion	None	Middle exclusion	None	Middle exclusion	None
**Accuracy**	69.5 ± 2	61.8 ± 2	70.8 ± 3	61.8 ± 1	71.9 ± 2	62.9 ± 3
**F1-score**	67.8 ± 2	59.1 ± 2	70.0 ± 2	60.7 ± 2	70.1 ± 2	60.6 ± 2
**AUPRC**	75.9 ± 1	66.6 ± 4	77.8 ± 1	65.2 ± 3	79.2 ± 1	67.7 ± 3
**MCC**	0.392	0.239	0.417	0.235	0.440	0.261
**AUROC**	75.2 ± 2	66.3 ± 2	77.3 ± 2	66.2 ± 2	78.1 ± 2	67.9 ± 3

The results in Table 4 show that middle exclusion boosted lncRNA localization prediction metrics on several model architectures. The boost could have had several causes: perhaps filtered training data was more informative for training or perhaps filtered test data was less challenging as a test. To evaluate these possibilities, we used the same data as above but with four data preparation protocols: no filtering, filtering all the data, filtering just the training subsets or filtering just the validation subsets. For this experiment, we used a neural network architecture and a GPU processor. Our model was a three-layer multi-layer perceptron (MLP) similar to DeepLncRNA (13) but with different hyperparameters; see Supplement 7. We implemented a middle-exclusion filter that could operate on each training partition or each validation partition during cross-validation. This allowed us to gather statistics under all four possible applications of middle exclusion.

The results are shown in Table 5. When middle exclusion was applied to all the lncRNA data, the metrics were comparable to published values, *e.g*. 77% AUROC in column A. Without middle exclusion, the metrics were substantially lower, *e.g*. 68% AUROC in column B. This comparison with an MLP demonstrates that middle exclusion does affect neural networks. Since middle exclusion has affected every machine learning architecture tested, we conclude that *the middle exclusion effect is a general problem that affects all model architectures*.

**Table 5.** Middle-exclusion effect on a neural network trained to classify lncRNA. The network architecture was an MLP. The features were k-mer profiles of RNA sequences. The data, features and training regimes were as in previous tables. The middle exclusion filter was applied to (**A**) all the data, (**B**) none of the data, (**C**) the training subsets only or (**D**) the validation subsets only

	A	B	C	D
Train filter: validation filter:	Middle excl. middle excl.	None none	Middle excl. none	None middle excl.
**Accuracy**	68.7 ± 2	62.2 ± 3	61.7 ± 1	70.9 ± 2
**F1**	63.0 ± 3	60.1 ± 4	61.3 ± 1	70.7 ± 2
**MCC**	0.390	0.254	0.236	0.419
**AUPRC**	78.7 ± 1	68.0 ± 3	66.4 ± 2	78.4 ± 2
**AUROC**	77.2 ± 1	67.8 ± 3	66.1 ± 1	77.6 ± 2

When middle exclusion was applied to the training sets only, the performance statistics remained low, e.g. 66% AUROC in column C of Table 5. But when middle exclusion was applied to the validation sets only, the performance statistics returned to their previous highs, e.g. 78% AUROC in column D. If filtering the training sets had improved model performance, then the column A results would surpass column D and the column C results would surpass column B, but they did not. Instead, it seems that filtering the training set had no effect on model performance. In contrast, filtering the validation sets clearly boosted the performance statistics, regardless of whether the training set was filtered, as the results in columns A and D surpassed those in columns B and C. A similar middle exclusion effect was seen when this experiment was repeated with mRNA data; see Supplementary Table S4. Also, when the lncRNA middle exclusion range was enlarged or reduced, the magnitude of the middle exclusion effect changed accordingly; see Supplementary Table S7. From this analysis, we conclude that *middle exclusion boosted the performance statistics, but not the actual performance, of these models*.

### Information leakage and other filters

We examined middle exclusion and other protocols used in the four studies, as summarized in Table 6. The RNAlight study includes two assessments for the model’s performance on the main dataset. Table 1 of their main paper shows 72% RNAlight accuracy on lncRNA from the withheld test subset. Their supplement presents an assessment based on averages over 5-fold cross-validation. Supplementary Table S3A of their supplement shows 69% RNAlight accuracy on lncRNA during cross validation. All the performance statistics were lower in cross-validation than in the final test. Since the test subset was chosen randomly, the difference could be attributed to the small sample size (n = 380) of the test set. The cross-validation results may be more representative, being based on more samples. Regardless, the test results were higher than predicted by cross-validation and this difference should have been noted in the main paper. The RNAlight study also included an assessment of the model on data derived from two Halo-seq experiments. However, that test set was filtered further, ‘removing redundant and bi-localized’ samples (16). As with middle exclusion, it would be helpful to see statistics with and without the filter.

**Table 6.** Summary of issues raised in this analysis. The four lncRNA subcellular localization studies are Gudenas & Wang (13), Lin *et al.* (14), Jeon *et al.* (15) and Yuan *et al.* (16)

Model name:	DeepLncRNA	lncLocator 2	TACOS	RNAlight
Authors:	Gudenas *et al.*	Lin *et al.*	Jeon *et al.*	Yuan *et al.*
Middle exclusion?	Yes	Yes	Yes	Yes
Data provided post-filtering?	Yes	No	n/a	Yes
Transcripts of same genes in train and test sets?	Likely	No	Likely	No
Combine non-coding mRNA with lncRNA?	No	Yes	Yes	No
Test set used for embedding?	n/a	Yes	No	n/a
Test set seen during training?	No	Yes	No	No
Test results exceeded cross-validation results	n/a	No	No	Yes

The DeepLncRNA study started with the construction of a dataset based on ENCODE experiments. The authors computed fold-change values for 18 000 transcripts. From the distribution, they extracted the most cytosolic and most nuclear quartiles and excluded the middle 50% of transcripts. “The dataset was then split into training, validation and testing sets using a randomized 70/15/15 percent split” (13). By this order of operations, the lower and upper threshold selection incorporated information from the test samples. An alternative approach would have split the transcripts first, then selected thresholds based on the training subset. Such alternate thresholds would probably be similar to the ones actually used, so any effect of this change may have been inconsequential, but it would have precluded information leakage from the test set.

However, the study may have suffered from another source of information leakage. Our analysis of the DeepLncRNA dataset found up to 40 transcripts of the same gene; see Supplementary Table S3. The text does not describe partitioning transcripts by gene and partition details were not provided, so the training/validation/testing split seems to be implemented per transcript. Since transcript isoforms of the same gene could share sequence and localization signals, the DeepLncRNA test set may have shared sequence with the training set. This potential form of leakage was precluded by the RNAlight study, which used one transcript per gene. It was made less likely by the lncLocator 2.0 study, which used one transcript per cluster after clustering all transcripts by sequence similarity. The lncLocator 2.0 benchmark data does contain multiple transcripts per gene, including genes with over 100 transcripts, in some cell lines. However, all transcripts of every gene appear to be grouped into the same subset correctly; see Supplement 4. The TACOS study used the lncLocator 2.0 data but with a modification: the combined training and validation subsets were down-sampled to achieve class balance. The sampled transcripts were shifted to the test subsets. This process may have led to genes with transcript representation in the training and testing subsets. In summary, assigning the various transcripts of a gene to multiple subsets risks information leakage. This form of leakage was scrupulously avoided in two of the four localization studies (14,16) but may have affected the other two (13,15).

Another potential source of information leakage appears in the lncLocator 2.0 study. Their model incorporated a GloVe word embedding (37). The pre-trained embedding was refined on the k-mer words extracted from the RNA sequences. Our reading of the main text and the source code indicates that the embedding was refined on the full RNA dataset prior to its partition into training and test subsets. If that was the case, the model may have incorporated k-mer combinations associated with the nuclear or cytoplasmic localization of specific RNAs in the test sets and that may have boosted the performance metrics.

Our analysis of the lncLocator 2.0 online data files indicated that non-coding mRNA transcript sequences were grouped with the lncRNA sequences for training and testing. This protocol may or may not affect the results, but it should be noted; see Supplement 4.

The lncLocator 2.0 source code repository includes Jupyter notebooks that ran the cross-validation experiments. These notebooks use all three data subsets simultaneously: training, validation and testing. During each fold of cross-validation, at each epoch, the code trains the model on the training subset and assesses it on the validation and testing subsets. Thus, the notebook provides a continuous preview of test performance while the model learns from the training data. This printed feedback could have had unintentional consequences by influencing the selection of hyperparameters such as the number of epochs for training. This suggests another way that information leakage may have occurred.

### A new benchmark

The results presented so far show that published lncRNA localization studies may have suffered from middle exclusion effects and information leakage effects that would have made their results unreliable indicators of model performance on uncharacterized lncRNA. To help this subfield of machine learning to make progress and measure progress carefully, we offer a simple model and a protocol for evaluating it on public data.

We measure performance on the lncATLAS data. The lncATLAS database provides CNRCI values for thousands of lncRNA genes as measured in 15 human cell lines by the ENCODE consortium. The database is a constant, having never been updated since its 2017 publication. Other datasets may become available over time and those may cover more genes, conditions, cell types and organisms, but results on the original lncATLAS data will provide a baseline for fair comparison. The approach used here could be applied to datasets that appear in the future.

We measure performance by two rounds of 5-fold cross-validation on the entire dataset. Note we do not reserve some portion as an unseen or independent test set. We believe the lncATLAS data has been so thoroughly studied that there is no longer an unseen portion. Instead, the use of cross validation provides variances with performance metrics. Other data sources could be used to build unseen and independent test sets.

Ideally, models would make predictions for any one of the 15 cell lines. In fact, the data is sparse overall and more so for some of the cell lines. This makes overfitting a pervasive problem. Therefore, we adopt the RNAlight approach of using mean values for training and prediction. Reliance on a statistic from multiple cell lines increases the number of genes whose data can be used for training. In a slight modification of RNAlight protocol, we use the log of the mean of the ratios rather than the mean of the log-odds, since this has a more direct interpretation. Following RNAlight protocol, we exclude the H1.hESC cell line because its CNRCI values correlate poorly with the other lines; see Supplementary Table S1. We stress that determinations like this should rely on training samples only, so as to preclude information leakage from test samples. In contrast to the RNAlight study, we do not apply the middle exclusion filter. Also, we do not filter genes whose CNRCI values indicate different localizations in different cell lines; the conflicting CNRCI values could not be detected on uncharacterized genes or lncRNA from a new cell line, so they are not used to filter our test set.

For training binary classifiers, the CNRCI values must be converted to binary labels with some threshold value. The lncLocator 2.0 and TACOS studies used threshold CNRCI = 0, which supports an obvious interpretation: since the CNRCIs are log-odds ratios, positive and negative values correspond to greater cytoplasmic and nuclear abundance, respectively. The DeepLncRNA and RNAlight studies both used thresholds within the nuclear range so as to generate balanced classes. We choose CNRCI = 0 as our threshold. We suggest that future studies may justify and use non-zero thresholds, but they should also assess their models using zero as a baseline. Because the zero threshold generates imbalanced classes and to avoid skewed statistics based on unequal classes, we balance the dataset by down-sampling the majority (nuclear) class.

The lncATLAS database characterizes gene expression by gene. It does not quantify or address which transcript(s) of a gene were detected. If different cell lines expressed different transcript isoforms of some gene, that cannot be determined from lncATLAS. The database does not include the transcriptome or genome reference sequence for any of the 15 cell lines. Thus, for prediction, it is necessary to use proxy sequences such as transcripts from the reference human genome. Following the RNAlight study, we rely on the canonical RNA sequence per gene, as identified within the GENCODE annotation. The lncLocator 2.0 dataset, which used all transcripts per gene, had the advantage of capturing more data and providing more training samples, but also had the risk of inflating the importance of genes with more transcripts. Also, about half its lncRNA samples were non-coding transcripts from protein-coding genes; the abundance of those isoforms may not be reflected by the lncATLAS CNRCI values.

We rely on a k-mer profile as the predictive feature per lncRNA, with k∈{3,4,5}. The k-mer profile feature was used by all the publications studied here, though some studies also incorporated other features. We leave it to future work to demonstrate whether additional features add predictive value.

Our benchmark model is the Microsoft implementation of LightGBM (20). This implementation is freely and publicly available for Python programmers. We use the default hyperparameter settings, leaving it for others to demonstrate whether other settings induce superior performance.

With threshold CNRCI = 0, the full dataset suffers from class imbalance, which can affect performance statistics such as accuracy, especially if the model over-predicts the majority class. Therefore, we down-sample the majority class. Using the balanced training and evaluation sets, our model’s performance statistics were 61% accuracy, 60% F1-score, 0.219 MCC, 66% AUPRC and 67% AUROC; see Supplementary Table S5. These statistics, though substantially lower than those shown by others, are meant to characterize model performance on unseen or uncharacterized lncRNAs.

## Discussion

We identified a machine learning protocol that we named middle exclusion. It consists of filtering the entire dataset, based on a function of the response variable, prior to partitioning the data into subsets for training, validation and testing. We showed that the protocol boosts performance statistics without boosting actual performance. We showed that metrics based on middle-exclusion experiments are not predictive of model performance on unseen, future or uncharacterized data.

**Figure 1.**
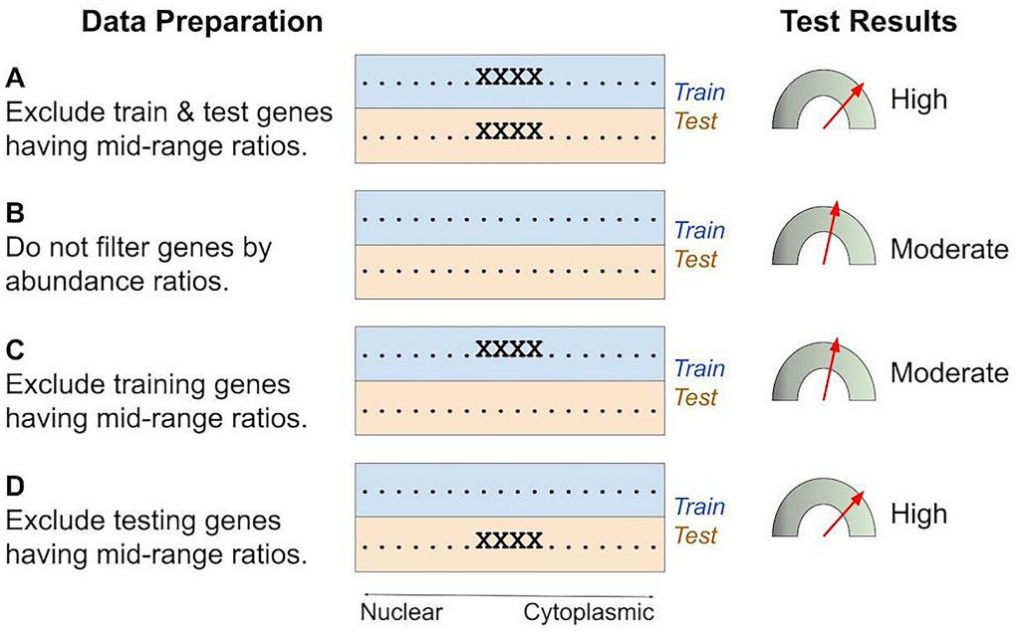
The middle exclusion effect. **Left**: Four published studies built models to predict lncRNA localization based on the few thousand genes that have known abundance ratios. All four studies filtered their datasets to remove intermediate values, as illustrated in row A. To explore the filter’s effects, we trained and tested machine learning models after filtering the training set only, the test set only, or both, or neither. **Right**: Filtering the test set boosted performance statistics (A and D versus B and C), but filtering the training set did not boost performance (C versus B). We argue that protocol A created unrealistic expectations of model performance and that protocol B should be used to characterize a model’s predictive utility on gene sets whose localizations have not already been measured.

We examined published studies of models trained to predict the nuclear-or-cytoplasmic localization of human lncRNAs. All these models used RNA sequences as features to predict the compartmental preference as measured by ENCODE and recorded in the lncATLAS database. All the studies reached a similar level of performance, with accuracy of approximately 72%. Our examination gives reasons to suspect that the assessment protocols could have boosted the performance statistics. Our results are summarized graphically in Figure 1. We conclude that the performance of each of the models was overstated. Knowing this could help the field by acknowledging that the problem is harder than currently perceived and that more work remains to be done and that investigators should be permitted to demonstrate incremental progress even if their performance statistics fall short of published values.

In retrospect, it was unexpected that the sequence-based machine learning methods would outperform another approach by a wide margin. Zuckerman & Ulitsky (33) built predictive models using experimentally derived features such as RNA splicing efficiency. These models had ‘typical precision and recall values of > 60%’ and explained 34% of the variance. Compared to the sequence-based results, their results present a harsher picture of how much localization is predictable.

Though middle exclusion was used in all four studies, it appears to be rarely used outside the narrow field of quantitative lncRNA subcellular localization. Our informal search found only two other uses of middle exclusion among recent machine learning publications in bioinformatics; see Supplement 9.

Our literature review noted other models that were assessed using the RNAlocate database. Those models were excluded from our analysis because they did not use middle exclusion (and cannot because the RNAlocate labels are discrete). They seem to outperform the ENCODE/lncATLAS-based models. For example, GraphLncLoc (29) achieved 83% AUROC on four-compartment classification. We caution that metrics based on RNAlocate are not directly comparable to metrics based on lncATLAS. Whereas lncATLAS quantifies nuclear versus cytoplasmic localization, RNAlocate recognizes two dozen compartments with extreme class imbalance. In addition, it is possible that the literature and thus RNAlocate are enriched for high-confidence and extreme localization cases.

The lncATLAS localizations for cell lines may not be predictive of localization in differentiated tissue or in living organisms. However, the machine learning classification task related to lncATLAS remains well defined. The database presents an abundance ratio for many genes in several cell lines. The classification task is to predict whether a given gene has greater nuclear or cytoplasmic abundance, in one cell line or across many.

Middle exclusion was applied in all four studies analysed. Middle exclusion has its merits. It can act as a noise filter by removing borderline and uncertain classifications. It was effectively used by the SEEKR study (12) to identify sequence features that were enriched at either extreme of the cytoplasmic-to-nuclear spectrum. However, as we have shown, middle exclusion can present an overly optimistic projection of model performance on uncharacterized data. Studies that filter their test sets by the attribute being predicted would serve readers well by presenting the results with and without the filter.

We identified possible instances of data leakage in the previously published lncRNA localization studies. In one example, the entire dataset had been used to parameterize part of a model prior to cross-validation. For cross-validation to generate accurate projections of future performance, we assert that this type of tuning should be repeated within each validation round, using only the data subset assigned to training for that round.

We presented a benchmark protocol with hopes that it will help the community to continue to make progress in this field. Our analysis focused on models of the lncATLAS data. Reliable models might help characterize the majority of lncRNA genes that are not quantified in this database. Unfortunately, the database has remained static since its creation. We are optimistic that imaging technologies, *e.g*. cell painting (38), will soon deliver quantitative localization data for more genes in more cell types under more conditions.

## Supplementary Material

lqae125_Supplemental_Files

## Data Availability

Source code for this project can be found at https://doi.org/10.5281/zenodo.10908398.
