# Supplementary material for: Evaluation of machine learning models that predict lncRNA subcellular localization
Source: NAR Genom Bioinform. 2024 Sep 18;6(3):lqae125. doi: 10.1093/nargab/lqae125 (PMC11409063; doi:10.1093/nargab/lqae125)
Supplement: lqae125_Supplemental_Files [file lqae125_supplemental_files.zip › Supplement.Jun17.pdf]

Supplement

Table of Contents

Supplement 1
- Supplement 1: lncATLAS 1
- Supplement 2: GENCODE 3
- Supplement 3: RNAlight and LightGBM 3
- Supplement 4: LncLocator 2.0 7
- Supplement 5: TACOS 9
- Supplement 6: DeepLncRNA 10
- Supplement 7: RF, GBM, SVM, MLP 11
- Supplement 8: Benchmark 13
- Supplement 9: Other Domains 14
- References for the Supplement 15

Supplement 1: lncATLAS

The lncATLAS database describes human genes in human cell lines (1). The initial release is still the only release. The full dataset was downloaded as a csv file[1] from the lncATLAS website[2] by clicking on Get Raw Data then Download All. The file provides one CNRCI value per gene per cell line for 15 human cell lines. The CNRCI is a log-odds ratio of cytoplasm abundance to nuclear abundance. The file includes mRNA and lncRNA genes (marked 'coding' and 'nc' respectively). Not all human genes are expressed in any given cell type, and not all human genes are represented in lncATLAS. For cell line K562 only, there are additional compartmental ratios besides CNRCI.

The lncATLAS database identifies genes by their Ensembl gene ID (prefix ENSG). It does not provide sequences or transcript IDs. However, it is possible to link genes to canonical transcript sequences using the Ensembl gene ID to search GENCODE. Not all genes listed in lncATLAS have corresponding sequences among the transcripts for genes mapped to chromosomes in the current release of GENCODE.

The following observations are supported by the lncATLAS_100.ipynb notebook. The database has CNRCI values for 6768 lncRNA genes. Of those, 4655 genes have a value in at least 2 cell lines, and none have a value in all 15 cell lines. The number of genes per cell line varies from 4923 for the H1.hESC cells to 582 for the IMR.90 cells. The mean CNRCI per cell line ranges from -0.5 for the H1.hESC cells to -1.8 for the

[1] lncATLAS_all_data_RCI.csv
[2] https://lncatlas.crg.eu/

SK.MEL.5 cells. Using the lncLocator2 thresholds (cytoplasmic: CNRCI>1, nuclear: CNRCI<-1, applied to each cell line), then the data removed by middle exclusion ranges from 29% to 52% of genes per cell line. Using the RNAlight thresholds (cytoplasmic: CNRCI>0, nuclear: CNRCI<-1, applied to mean of 14 cell lines), the data removed by middle exclusion is 39% of genes with values in those cell lines.

The RNAlight study used mean CNRCI "excluding H1 cell line due to its low correlation to other cell lines, data not shown". We computed pairwise Pearson correlations of CNRCI values between genes in common between each cell line pair, and the average correlation between each cell line and the other cell lines, for the lncRNA and separately for the mRNA data. The results were computed in notebook CellLineCorrelation_105 and are summarized in **Table S1**. Among the lncRNA values, only H1.hESC is more than 3 standard deviations below the mean. Among the mRNA data, no cell line reaches that mark, though NCI.H460 comes closest.

Cell Line	lncRNA	mRNA
A549	0.76	0.74
GM12878	0.72	0.75
H1.hESC	0.50	0.62
HT1080	0.77	0.74
HUVEC	0.80	0.79
HeLa.S3	0.75	0.79
HepG2	0.78	0.79
IMR.90	0.80	0.79
K562	0.74	0.78
MCF.7	0.77	0.78
NCI.H460	0.63	0.59
NHEK	0.75	0.73
SK.MEL.5	0.72	0.71
SK.N.DZ	0.73	0.72
SK.N.SH	0.77	0.78

Table S1. Correlations in lncATLAS data. Each row shows the average correlation between one cell line and 14 others. The lncRNA and mRNA data were analyzed separately. For each pair of

cell lines, the Pearson correlation was computed by comparing the CNRCI values for genes having a numeric CNRCI in each cell line.

Supplement 2: GENCODE

The GENCODE database (2) organizes DNA and RNA sequences and annotations. The Ensembl database (3) is a source for much of the contents. Text files were downloaded from the main website[3] by clicking How to Access Data, then Human FTP site, then Release 44 or 45[4]. We downloaded the sequence file[5] in FASTA format and the annotation file[6] in GFF format. We used a script[7] to list the canonical transcript ID per gene based on a tag in the GENCODE annotations. (Ensembl documentation says various criteria affect the selection of canonical transcript per mRNA gene, but maximum genomic span is the only criteria used to select the canonical transcript per lncRNA gene[8].)

Supplement 3: RNAlight and LightGBM

RNAlight is a predictive model (4). This study used the average CNRCI per gene across 14 cell lines (excluding cell line H1.hESC). In this study, genes having CNRCI < -2 were labeled cytoplasmic, and genes having CNRCI > 0 were labeled nuclear. Genes with intermediate values were excluded. These thresholds would retain 1525 cytoplasmic and 1983 nuclear genes, and excludes 39% of genes; see notebook lncATLAS_100.

The RNAlight lncRNA predictions file[9] was obtained from the RNAlight source code repository. The text file contains one line per gene for 16153 genes. Each line contains the columns: Ensembl transcript ID, common gene name, RNA sequence of the canonical transcript, RNAlight predicted label of cytoplasmic or nuclear (zero or one, respectively), and RNAlight predicted probability of being nuclear (zero to one). We analyzed the data in the RNAlight_101.ipynb notebook. Most of the transcripts listed are not characterized in lncATLAS and a few are not in the current GENCODE, but we matched 5313 transcripts to genes with CNRCI values in lncATLAS. Of those, 2091 have middle values, while 3222 have extreme values, using the RNAlight approach of computing the mean CNRCI across 14 cell lines and labeling genes as cytoplasmic or nuclear if their mean is greater than 0 or less than -2, respectively. Comparing RNAlight

[3] https://www.gencodegenes.org/
[4] https://ftp.ebi.ac.uk/pub/databases/gencode/Gencode_human/release_45/
[5] gencode.v45.lncRNA_transcripts.fa.gz
[6] gencode.v45.long_noncoding_RNAs.gff3.gz
[7] get_canonical_transcript_ids.45.sh
[8] https://useast.ensembl.org/info/genome/genebuild/canonical.html
[9] https://github.com/YangLab/RNAlight/blob/main/Light_score_diverse_RNA/lncRNA_whole_genome/Whole_genome_lncRNA_predict_df.tsv

predictions to the mean CNRCI values, these predictions were 96% accurate on the extreme genes. It seems likely that the predictions are derived from a model that had been retrained on all the extreme cases, enabling near perfect accuracy on this set. According to its publication, RNAlight had 72% accuracy on examples that had been withheld from training. The middle genes would not have been used for training, so it was informative to measure the accuracy of predictions on these. With these data, we observed 55% accuracy among the RNAlight predictions on genes with mid-range CNRCI values; see main paper, **Table 2**, row A. This test used the threshold CNRCI = -1 because -1 is the midpoint of the [-2, 0] middle range used by RNAlight and accuracy declined at the other thresholds tested among {-2.0, -1.5, -1.0, -0.5, 0.0}; see notebook RNAlight_101.

The RNAlight dataset is given in supplemental files. The main dataset was generated from three sources: the lncATLAS collection of ENCODE experiments, plus data from CeFra-seq experiments and APEX-Seq experiments. An independent test set was generated from Halo-seq experiments. The data files provided contain the transcripts used for training and testing. There are separate nuclear and cytoplasmic files[10]. The files do not contain the transcripts that were filtered by middle exclusion, or the transcripts that were filtered for being “redundant” or “bi-localized.” Having only the post-filtered data, it was unfeasible for us to reconstruct the entire dataset for filtered vs. unfiltered experiments. Instead, we used the lncRNA data from lncATLAS and processed it according to RNAlight methods.

For the RNAlight and LightGBM comparisons, we relied on the mean $\log_2$ CNRCI across 14 cell lines, excluding the H1.hESC cell line. (The RNAlight methods section says the exclusion of H1.hESC left 13 cell lines, but we assume the remaining 14 were used.) For the RNAlight and LightGBM comparisons, we incorporated RNAlight source code into our Jupyter notebooks with as few changes as possible. We note that the RNAlight code computes means of logs (whereas logs of means of antilogs might be easier to interpret). We note that the RNAlight code only counts non-overlapping k-mers (by relying on the count() method of the Python str class), so it would count only 1 instance of 3-mer ‘AAA’ within sequence ‘AAAA’. The RNAlight approach to transcripts uses one transcript per gene, specifically the one marked ‘Ensembl_canonical’ in GENCODE annotations. (As previously noted, the Ensembl canonical transcript of a lncRNA gene is the one with the maximum genomic span[11].)

The RNAlight model is an instance of LightGBM (5). We tested LightGBM on lncATLAS data processed by RNAlight protocols by computing the mean CNRCI over 14 cell lines and using (-2,0) as the CNRCI range for middle exclusion; see notebook RNAlight_123. When not using middle exclusion, we used threshold CNRCI = -1 to label genes as nuclear or cytoplasmic. We implemented class balance by randomly

[10] 02_lncRNA_info_nuc_transcript.tsv and 02_lncRNA_info_cyto_transcript.tsv
[11] https://useast.ensembl.org/info/genome/genebuild/canonical.html

down-sampling the majority class. We applied RNAlight code to generate k-mer profiles of the GENCODE canonical RNA transcript per gene. The model's objective was to classify each lncRNA as nuclear or cytoplasmic given only its k-mer profile. We evaluated models by 5-fold cross-validation.

The RNAlight model is an instance of LightGBM (5) with hyperparameters optimized on the RNAlight training set. It was not feasible to reproduce the RNAlight settings exactly because the settings were given in a pickle file that has dependencies on historic software versions. However, we ran the RNAlight code that performs a grid search for optimal hyperparameter settings. This used the same Microsoft implementation[12] of LightGBM though the most recent version, and with our training data derived from lncATLAS. The optimized settings are shown in **Table S2**. Most optimized settings are similar to the defaults, with a striking difference being n_estimators, which was 100 by default but was optimized to 2200. This change was observed to greatly extend training time. We therefore used LightGBM with default hyperparameters for this test.

Hyperparameter ranges explored	"learning_rate":[0.1, 0.05, 0.02, 0.01],<br>"num_leaves": range(10,36,5),<br>"max_depth" : [2,3,4,5,10,20,40,50],<br>"min_child_samples": range(1, 45, 2),<br>"colsample_bytree" : [i / 10 for i in range(2,11)],<br>"metric" : ["binary_logloss"],<br>"n_estimators" : range(100,2500,100),<br>"subsample" : [i / 10 for i in range(2, 11)],<br>"subsample_freq" : [0, 1, 2],<br>"reg_alpha" : [0, 0.001, 0.005, 0.01, 0.1],<br>"reg_lambda" : [0, 0.001, 0.005, 0.01, 0.1]
LightGBM default values	'boosting_type': 'gbdt',<br>'class_weight': None,<br>'colsample_bytree': 1.0,<br>'importance_type': 'split',<br>'learning_rate': 0.1,<br>'max_depth': -1,<br>'min_child_samples': 20,<br>'min_child_weight': 0.001,<br>'min_split_gain': 0.0,<br>'n_estimators': 100,<br>'n_jobs': None,<br>'num_leaves': 31,<br>'objective': None,

[12] https://github.com/microsoft/LightGBM/releases

	'random_state': None,<br>'reg_alpha': 0.0,<br>'reg_lambda': 0.0,<br>'subsample': 1.0,<br>'subsample_for_bin': 200000,<br>'subsample_freq': 0
Hyperparameters values selected	'boosting_type': 'gbdt',<br>'class_weight': None,<br>'colsample_bytree': 0.5,<br>'importance_type': 'split',<br>'learning_rate': 0.01,<br>'max_depth': 40,<br>'min_child_samples': 9,<br>'min_child_weight': 0.001,<br>'min_split_gain': 0.0,<br>'n_estimators': 2200,<br>'n_jobs': 1,<br>'num_leaves': 35,<br>'objective': 'binary',<br>'random_state': 100,<br>'reg_alpha': 0.005,<br>'reg_lambda': 0,<br>'subsample': 0.6,<br>'subsample_for_bin': 200000,<br>'subsample_freq': 1,<br>'metric': 'binary_logloss'

Table S2. An exploration of the LightGBM hyperparameters optimized by RNAlight. Top: the hyperparameters and ranges explored by the grid search implemented in RNAlight code. Middle: the LightGBM default values. Bottom: the RNAlight grid search was replicated in our notebook RNAlight_122 after which the model was saved and explored in notebook RNAlight_125. The optimized values were extracted by invoking model.get_params().

To evaluate LightGBM, and subsequent models, we adapted code from the RNAlight repository. The code uses a random number generator for reproducibility. It makes a random 90:10 train:test partition of the genes. During each fold of 5-fold cross-validation on the train subset, the code makes another random partition of the data, with one portion used for training and the other used for validation. (The average performance across validation sets could be used to predict performance on the withheld test set. However, in this study, we report the validation statistics without using the withheld test set.) Our experiments used 2 rounds of 5-fold cross-validation so each mean and standard deviation reflects 10 trials. To help us run the code with and without

middle exclusion, we generated different versions of the input files; see notebook RNAlight_123. Like the RNAlight predictions file, these were split into separate files for nuclear and cytoplasmic genes, according to the mean CNRCI across 14 cell lines of lncATLAS data.

The LightGBM model was first evaluated by cross validation using the RNAlight data (which lacks any genes with middle-range CNRCI values). The performance metrics were moderate, *e.g.* 73% AUROC. The same model was then evaluated by cross validation on our lncATLAS dataset, presented with and without middle exclusion. When the middle exclusion filter was applied to the data, the model's performance statistics were high, *e.g.* 78% AUROC. The statistics for the same model without middle exclusion were low, *e.g.* 66% AUROC. See main paper, **Table 3**, and notebook RNAlight_127.

For completeness, we ran the experiment under various conditions: using either lncRNA or mRNA data; using data from either RNAlight or lncATLAS; using all the data or sampling down the major class to achieve class balance; and applying or not applying a middle exclusion filter; see **Table S6** and notebooks RNAlight_127, _128, _137, _138. The following trends were observed. Balancing the lncATLAS data altered the F1 score (as well as precision and recall, not shown). The RNAlight data are nearly class balanced so balancing them had less effect. The LightGBM model performed slightly better with optimized parameters than with default parameters. The model performed better on mRNA than on lncRNA; the differences were small using RNAlight data but larger using lncATLAS data. (This trend was also seen using an MLP model; see Supplement 7.) Middle exclusion boosted the performance statistics in all cases.

Supplement 4: LncLocator 2.0

LncLocator2 is a predictive model (6). In this study, genes having CNRCI < -1 were labeled nuclear, and genes having CNRCI > +1 were labeled cytoplasmic. These thresholds would exclude intermediate values, affecting 29% to 52% of genes per cell line; see notebook lncATLAS_100.

We obtained source code the authors' code repository[13] and a benchmark dataset[14] from their website[15].

The benchmark dataset includes many sequence files in csv format. For each cell line, there are separate files containing the training, validation, and testing subsets. These files contain the gene and transcript IDs, as well as the sequences. There are many genes with multiple transcripts. For example, in the HeLa cell line files, these 5 genes have over 100 transcripts each: ENSG00000227036, ENSG00000242086, ENSG00000215386, ENSG00000249859, and ENSG00000179818. However, the

[13] https://github.com/Yang-J-LIN/lncLocator2
[14] benchmark.zip from http://www.csbio.sjtu.edu.cn/bioinf/lncLocator2/Data.htm
[15] http://www.csbio.sjtu.edu.cn/bioinf/lncLocator2/

multiple transcripts for each gene are co-located to one of the three subsets. We found no genes represented in more than one subset for one cell line.

The benchmark dataset represents many (probably all) transcripts per gene. All transcripts from lncRNA genes are in files named “lncRNA.csv”, but these files also contain transcripts from protein-coding genes. The transcripts from protein-coding genes make up about half the sequences in the lncRNA files *e.g.* 24320 out of 42965 for the H1.hESC cell line. An example is provided by gene ENSG00000004866 (common name ST7), for which the Ensembl web site displays 19 protein-coding transcripts and 7 non-coding isoforms. In the lncLocator 2.0 benchmark dataset, the 19 coding transcripts appear in “protein_coding.csv” files, while the 7 non-coding isoforms appear in “lncRNA.csv” files for each cell line. All the entries for this gene necessarily carry the same CNRCI value because lncATLAS characterizes genes not transcripts. The H1.hESC CNRCI value for ENSG00000004866 is 0.538593 (slightly cytoplasmic). Since mRNA is generally expressed at higher levels than lncRNA, and since non-coding mRNAs are likely expressed at lower levels than their protein-coding isoforms, it seems likely that this CNRCI value from lncATLAS was measured on the protein-coding mRNA molecules. It may be problematic to associate that value with each of the non-coding isoforms and use them for training and evaluating the lncRNA localization classifier.

The lncLocator2 model source code includes the embedding layer, already trained on RNA sequences. The source code uses CNRCI=0 as the nuclear-vs-cytoplasmic threshold. It has parameters that enable or disable middle exclusion on each of the data subsets: training, dev a.k.a. validation, and testing. (With a code tweak, these can be set via the command line.) With middle exclusion enabled, the code excludes genes with CNRCI values in the [-1,+1] range. The code runs 100 epochs of training by default. After each epoch, it computes performance statistics and logs a report for each data subset: training, validation, testing.

The lncLocator2 publication highlights cell line H1.hESC, calling it “illustrative” and “baseline” and using it in its Table 4 to compare models. That table shows AUROC=0.8472 for lncLocator2. We used the same cell line for a test of performance on two test sets. This experiment exploited existing code that made the middle-exclusion filter optional. However, it required minor code changes to invert the middle-exclusion filter and to enable or disable it from the command line. The first set was similar to the middle-exclusion tests used in the publication, as it contained only genes with H1.hESC CNRCI values outside the [-1,+1] range. On this set, we saw AUROC 0.8282 and 75% accuracy on the test set; see the LL2_H1_YY notebook and log file. The second set was the complement, having only genes with values inside the [-1,+1] range. On this set, we saw AUROC 0.5921 and 59% accuracy; see the LL2_H1_middle notebook and csv file. This result appears in the main paper, **Table 2**, row B. Thus, performance on the extreme genes was substantially better than performance in intermediate genes.

Supplement 5: TACOS

TACOS is a web server[16] that predicts nuclear or cytoplasmic localization given an RNA sequence and the name of a cell line (7).

The TACOS project adopted the lncLocator1 data and its thresholds, such that middle exclusion affected genes with CNRCI values in the range [-1,+1]. The TACOS study used the lncLocator2 data with this modification: the combined training + validation subsets were down-sampled to achieve class balance. Critically, the “leftover” transcripts were shifted to the test subsets per cell line. The text does not describe shifting by gene. If the transcripts were sampled and shifted randomly, then some genes may have gained representation in the training, validation, and test subsets. This possibility is made critical by the observation that the lncLocator2 data contains multiple transcripts per gene. In an extreme example, gene ENSG00000179818 has 238 examples in the lncLocator2 validation set for the HeLa cell line.

The TACOS server supports 10 of the 15 cell lines in lncATLAS. For each of those 10 cell lines, we generated a FASTA file that contained 100 randomly selected sequences for which the lncATLAS CNRCI was between -1 and +1 and for which the canonical transcript sequence was available in GENCODE; see notebook TACOS_201. Each file was uploaded manually to the TACOS server, and the corresponding cell line was selected from the pull-down menu. Each generated CSV file was saved to disk. The 10 results files contained three fields per line: FASTA defline, predicted localization (either ‘Nucleus’ or ‘Cytoplasm’), and a probability (between 0 and 1). We considered ‘Nucleus’ to be a correct prediction if the true CNRCI was zero or below, and ‘Cytoplasm’ to be a correct prediction if the true CNRCI was zero or above; see notebook TACOS_202. The accuracy of these 10*100=1000 predictions was 56.15%. This value is reported in the main paper, **Table 2**. This value is substantially lower than what is claimed in the publication, and the reason appears to be our use of the mid-range CNRCI values that were excluded from the TACOS publication.

In our tests with middle values, the maximum accuracy per cell line was 67% on H1.hESC. A possible explanation is that this cell line offers the largest training set, having the most CNRCI values of any cell line in lncATLAS. Also, the TACOS server performed better on genes whose CNRCI was not middle-valued. Using 10 random genes with CNRCI > 1 and 10 with CNRCI < 1 from each of the 10 cell lines, we observed 85% accuracy in the positive range and 89% in the negative range (data not shown). Both of these values exceed what is claimed in the publication, suggesting that most or all those genes had been included in the TACOS training sets.

[16] https://balalab-skku.org/TACOS

Supplement 6: DeepLncRNA

The DeepLncRNA (8) dataset was provided in a supplement to the publication. The supplemental spreadsheet provides one transcript per row and one feature per column. The number of rows, 8678, matches the sum of 4380 cytosolic and 4298 nuclear transcripts reported in the main text. Thus, this supplemental file appears to lack data for over 8000 transcripts, likely the ones filtered by middle exclusion. Reconstructing features and abundance labels for the omitted transcripts would be challenging; this study did not use lncATLAS data but rather on computed abundance ratios directly from raw ENCODE RNA-seq data. The process described in the supplemental methods is complex. Thus, it was not feasible for us to test models on the DeepLncRNA dataset with and without middle exclusion.

Within the supplementary spreadsheet, the features described in columns include transcript length, k-mer counts, the chromosome that hosts the gene, and whether the gene is intergenic, sense, or antisense relative to protein-coding genes. The first column is labeled “Ensembl Gene ID” although it contains Ensembl transcript IDs, as indicated by their “ENST” prefixes. We related these transcript IDs to gene IDs in GENCODE and detected multiple transcripts per gene. The most extreme example, one gene with 40 transcripts, is shown in **Table S3**.

**Transcript ID used by DeepLncRNA**	**Gene ID from GENCODE**
ENST00000366234	ENSG00000179818
ENST00000413436	ENSG00000179818
ENST00000416395	ENSG00000179818
ENST00000419542	ENSG00000179818
ENST00000421255	ENSG00000179818
ENST00000422515	ENSG00000179818
ENST00000432604	ENSG00000179818
ENST00000439892	ENSG00000179818
ENST00000442040	ENSG00000179818
ENST00000457076	ENSG00000179818
ENST00000458686	ENSG00000179818
ENST00000597318	ENSG00000179818
ENST00000598586	ENSG00000179818
ENST00000599427	ENSG00000179818
ENST00000600002	ENSG00000179818
ENST00000601395	ENSG00000179818
ENST00000602091	ENSG00000179818
ENST00000603347	ENSG00000179818
ENST00000609075	ENSG00000179818
ENST00000613944	ENSG00000179818
ENST00000625227	ENSG00000179818
ENST00000625257	ENSG00000179818
ENST00000626440	ENSG00000179818
ENST00000626509	ENSG00000179818
ENST00000626558	ENSG00000179818

ENST00000626683	ENSG00000179818
ENST00000626842	ENSG00000179818
ENST00000627325	ENSG00000179818
ENST00000627540	ENSG00000179818
ENST00000627630	ENSG00000179818
ENST00000628308	ENSG00000179818
ENST00000628455	ENSG00000179818
ENST00000628667	ENSG00000179818
ENST00000629188	ENSG00000179818
ENST00000629825	ENSG00000179818
ENST00000629943	ENSG00000179818
ENST00000630520	ENSG00000179818
ENST00000630759	ENSG00000179818
ENST00000630975	ENSG00000179818
ENST00000631110	ENSG00000179818

Table S3. Multiple transcripts of the same gene found in the DeepLncRNA supplemental data file.

The DeepLncRNA model was built with a commercial software package, H2O. The specific version is no longer supported. This made it challenging to reconstruct the model. We implemented a similar architecture (multi-layer perceptron) with the open-source Tensorflow library. We tested our model on the lncATLAS data. The results in notebooks MLP_201 and MLP_202 are described in the MLP section of this supplement.

Supplement 7: RF, GBM, SVM, MLP

Three machine learning models were trained and tested with and without middle exclusion on lncRNA data. Each test used the same data, feature extraction, and thresholds as the LightGBM experiments; Supplement 3. The data consists of the subset of transcripts that are in the RNAlight predictions file and could be aligned with GENCODE and LncATLAS. For the middle exclusion test, genes with CNRCI in range [-2,0] were excluded. For the test without middle exclusion, CNRCI = -1 was used as the nuclear-or-cytoplasmic threshold. The models ran on a high-RAM CPU cloud computer (no GPU) on Google CoLab; see notebooks RF_201, GBM_201, SVM_201. Each notebook ran several tests but we relied on the tests labeled as our lncATLAS training set (based on the RNAlight predictions file) rebalanced (by randomly sampling down the majority class). Statistics based on the balanced classes were preferred because the accuracy and F1 statistics depend on which class is declared positive when the classes are of unequal size, even though the down-sampled training sets were smaller. The results (main paper, **Table 4**) show that middle exclusion boosted lncRNA localization prediction metrics on each of these model architectures.

Next, a neural network was tested for a middle exclusion effect. The overall architecture was that of a multi-layer perceptron (MLP), as was DeepLncRNA (8).

These experiments ran on cloud computers with a GPU (V100, A100, or L4 depending on availability). The model was tested under four regimes: middle exclusion applied to the entire dataset or no middle exclusion (notebook MLP_201), then middle exclusion applied only to the training set or only to the test set (notebook MLP_202).

Four sets of performance statistics are shown in the main paper, **Table 5**. Two sets are higher (having about 70% accuracy) than the other two (having about 62% accuracy). The higher-performance results involved validation sets that had been filtered by middle exclusion; between these, filtering the training set made no perceptible difference. The lower-performance results involved validation sets that had not been filtered; between these, filtering the training set made no perceptible difference. These results combine to show that middle exclusion on the training sets did not make the models more performant, but filtering the validation sets boosted their performance statistics.

The lncATLAS database provides subcellular localization values for mRNA as well as lncRNA. Though mRNA must reach the cytoplasm to fulfill its protein-coding function, lncATLAS contains a wide range of CNRCI values (from -10.3 to +5.58) for mRNA. Yuan *et al*. trained and evaluated the RNAlight model on the mRNA and lncRNA data from lncATLAS; see tables 1 and 2 in that paper. Their mRNA and lncRNA performances were similar by accuracy (72%, 73%), MCC (0.45, 0.45), and AUROC (80%, 78%), but different by F1 score (66%, 74%). Their statistics reflect tests on their dataset, which includes values from lncATLAS and two other sources, filtered by middle exclusion.

We used the lncATLAS mRNA data to repeat our comparison of four different applications of a middle exclusion filter: on the training set only, on the test set only, on neither, and on both. On lncRNA, filtering the training set did not enhance the MLP model, but filtering the test set did boost the statistics; see main paper, **Table 5**. A similar trend was seen with the MLP and mRNA data; see **Table S4, Table S7**, and notebooks MLP_201, _202, _203, _204. The following trends were observed. The MLP performance on lncATLAS mRNA data with middle exclusion (column A) exceeded what was reported for RNAlight on RNAlight mRNA data; this trend was also seen with the LightGBM model; see Supplement 3. Filtering the test set boosted MLP statistics (columns A and D) regardless of whether the training set was filtered, but filtering the training set did not improve MLP performance (columns B and C). Filtering the training set had a slight negative effect, possibly by reducing the number of training samples. Overall, filtering the data had a large positive effect on the model’s statistics but no positive effect on the model’s performance, with mRNA as with lncRNA.

	A	B	C	D
**Train filter:**<br>**Validation filter:**	middle excl.<br>middle excl.	none<br>none	middle excl.<br>none	none<br>middle excl.
**Accuracy**	80.5 ±2	70.7 ±1	64.9 ±2	80.3 ±2
**F1**	80.3 ±2	71.9 ±1	66.5 ±2	79.1 ±1
**MCC**	0.610	0.416	0.301	0.612
**AUPRC**	85.4 ±2	73.3 ±1	67.0 ±2	86.2 ±3
**AUROC**	86.9 ±2	77.0 ±1	70.1 ±1	87.9 ±2

Table S4. Middle-exclusion effect on a neural network trained to classify mRNA. The network architecture was a multi-layer perceptron (MLP). The features were k-mer profiles of RNA sequences. The data, features, and training regimes were as in previous tables. The middle exclusion filter was applied to (A) all the data, (B) none of the data, (C) the training subsets only, or (D) the validation subsets only.

The MLP experiment was repeated while altering the size of the middle exclusion range. The observed performance boost attributable to middle exclusion grew as the middle exclusion range grew. For example, the accuracy of the MLP with middle exclusion on lncRNA was 66.6% or 68.7% or 72.1% for the middle exclusion ranges [-1.5, -0.5] or [-2.0, 0.0] or [-2.5, +0.5], respectively, while the accuracy without middle exclusion remained 62.2%; see Table S7.

Supplement 8: Benchmark

A benchmark dataset was created in notebook Benchmark_100. The data is derived from the lncATLAS and GENCODE databases. Following the RNAlight protocols, each gene is characterized by its mean CNRCI across 14 cell lines (excluding the 15th cell line, H1.hESC). Each mean is computed as a mean ratio; since each CNRCI is a base-2 log-odds ratio for each gene we compute the antilogs of its CNRCI values, then compute the mean of those, and finally take the base-2 log. Formally, let $\mathbf{L_g}$ be the number of CNRCI values in lncATLAS for gene **g**. Each L_g is at least 1 and at most 14, since we ignore non-numeric values and we exclude one of the 15 cell lines. Let $\mathbf{CNRCI_{gi}}$ be the **i**th CNRCI value for gene **g**. Then the mean value for gene g is computed as:

$$M_g = log_2\left(\left[\sum_{i=1}^{L_g} 2^{(CNRCI_{gi})}\right] / L_g\right)$$

A benchmark model was trained and evaluated in notebook Benchmark_101. The code adapted from RNAlight performs feature extraction and cross-validation. Following lncLocator2, zero is used as the CNRCI threshold to label a gene as cytoplasmic (positive CNRCI) or nuclear (zero or negative CNRCI).

The performance statistics are shown in **Table S5**. A LightGBM model was evaluated by 2 rounds of 5-fold cross-validation. An initial run is performed using all the data, but the class imbalance skews the results. F1-score and AUPRC are affected because they use precision and recall, which are directional statistics whose values depend on which class is considered the positive class. Accuracy is also affected if the model over-predicts the majority class. Therefore, the majority class (nuclear genes) was randomly down-sampled to achieve class balance. Unfortunately, down-sampling reduces the size of the training set and might degrade performance. Nevertheless, the balanced subset was used for the proposed benchmark since it is the most predictive of model performance on unseen data.

	**lncATLAS Data (imbalanced)**	**Benchmark Subset (balanced)**
**Accuracy**	67.1 ±2	60.9 ±2
**F1**	76.1 ±3	60.0 ±3
**MCC**	0.260	0.219
**AUPRC**	78.0 ±1	65.8 ±3
**AUROC**	68.6 ±1	66.7 ±1

Table S5. Cross-validation performance statistics for the proposed benchmark model on the proposed benchmark dataset. Each gene was characterized by its mean CNRCI with positive values labeled cytoplasmic and all others nuclear. The lncATLAS dataset was balanced by down-sampling the majority (nuclear) class. The balanced set had the disadvantage of presenting fewer samples for training, but the advantage of not rewarding models that overpredict the majority class.

Supplement 9: Other Domains

As reported in the main paper, our literature search found four machine learning publications in which the model was measured on quantitative data filtered by a middle exclusion filter on the response variable. We also searched for recent applications of middle exclusion to other areas of bioinformatics. We found the two publications described below. Although this search could not be done exhaustively, middle exclusion appears uncommon in bioinformatics.

Ashraf *et al.* (9) used machine learning to predict the bioactivity of one class of drug candidates designed to treat COVID-19. They relied on a public dataset containing the half-maximal inhibitory concentration (IC50) of various compounds. They grouped the data into three classes using two thresholds: class Active for IC50 < 1K nM, class Intermediate for 1K ≤ IC50 < 10K nM, and class Inactive for IC50 > 10K nM. They excluded the intermediate cases from their study. They trained models on chemical structure descriptors and evaluated them as binary classifiers *i.e.* how well they could infer the active or inactive label from the descriptors. If the test data were pre-filtered by the very attribute that the models were designed to predict, then the performance statistics are based on middle exclusion.

Kakati et al. (10) used machine learning to classify genes as up or down regulated given RNA-seq data from diseased and normal cells for various cancer types. Their model achieved up to 100% in cross-validation and up to 99% on the reserved test sets from the various cancers. It appears that the dataset “Q” from which the test set “T3” was drawn had been filtered to retain the significantly differentially expressed genes (“SDEGs”) defined as being in the upper and lower quartiles of the differential expression range. If so, the accuracy statistics are based on middle exclusion.

References for the Supplement

1. Mas-Ponte,D., Carlevaro-Fita,J., Palumbo,E., Hermoso Pulido,T., Guigo,R. and Johnson,R. (2017) LncATLAS database for subcellular localization of long noncoding RNAs. *RNA*, **23**, 1080–1087.

2. Frankish,A., Carbonell-Sala,S., Diekhans,M., Jungreis,I., Loveland,J.E., Mudge,J.M., Sisu,C., Wright,J.C., Arnan,C., Barnes,I., *et al.* (2023) GENCODE: reference annotation for the human and mouse genomes in 2023. *Nucleic Acids Res.*, **51**, D942–D949.

3. Cunningham,F., Allen,J.E., Allen,J., Alvarez-Jarreta,J., Amode,M.R., Armean,I.M., Austine-Orimoloye,O., Azov,A.G., Barnes,I., Bennett,R., *et al.* (2022) Ensembl 2022. *Nucleic Acids Res.*, **50**, D988–D995.

4. Yuan,G.-H., Wang,Y., Wang,G.-Z. and Yang,L. (2023) RNAlight: a machine learning model to identify nucleotide features determining RNA subcellular localization. *Brief. Bioinformatics*, **24**.

5. Ke,G., Meng,Q., Finley,T., Wang,T., Chen,W., Ma,W., Ye,Q. and Liu,T.-Y. (2017) Lightgbm: A highly efficient gradient boosting decision tree. *Advances in neural information processing systems*, **30**.

6. Lin,Y., Pan,X. and Shen,H.-B. (2021) lncLocator 2.0: a cell-line-specific subcellular localization predictor for long non-coding RNAs with interpretable deep learning. *Bioinformatics*, **37**, 2308–2316.

7. Jeon,Y.-J., Hasan,M.M., Park,H.W., Lee,K.W. and Manavalan,B. (2022) TACOS: a novel approach for accurate prediction of cell-specific long noncoding RNAs subcellular

localization. *Brief. Bioinformatics*, **23**.

8. Gudenas,B.L. and Wang,L. (2018) Prediction of LncRNA Subcellular Localization with Deep Learning from Sequence Features. *Sci. Rep.*, **8**, 16385.

9. Ashraf,F.B., Akter,S., Mumu,S.H., Islam,M.U. and Uddin,J. (2023) Bio-activity prediction of drug candidate compounds targeting SARS-Cov-2 using machine learning approaches. *PLoS ONE*, **18**, e0288053.

10. Kakati,T., Bhattacharyya,D.K., Kalita,J.K. and Norden-Krichmar,T.M. (2022) DEGnext: classification of differentially expressed genes from RNA-seq data using a convolutional neural network with transfer learning. *BMC Bioinformatics*, **23**, 17.
